# Exploring Professional Autonomy Among Palestinian Nurses: A Comprehensive Scoping Review of Determinants, Barriers and Clinical Practice Implications

**DOI:** 10.1002/nop2.70652

**Published:** 2026-06-17

**Authors:** Ibrahim Aqtam, Saqr Alkorom, Mustafa Shouli

**Affiliations:** ^1^ Department of Nursing Nablus University for Vocational and Technical Education Nablus Palestine

**Keywords:** clinical autonomy, conflict settings, determinants, healthcare system, nurses, nursing policy, nursing practice, Palestine, patient safety, professional autonomy

## Abstract

**Aim:**

To systematically map the determinants, expressions and implications of nurses' clinical autonomy in Palestinian healthcare settings.

**Background/Introduction:**

Nurses' autonomy is crucial for quality care, job satisfaction and patient safety. In Palestine, the healthcare system operates under unique political instability and resource constraints, yet a comprehensive synthesis of nursing autonomy in this context is lacking.

**Methods:**

A scoping review was conducted following PRISMA‐ScR guidelines. PubMed, Scopus, CINAHL, PsycINFO, Google Scholar, ProQuest, Al‐Manhal and Arab World Research Source were searched for literature published between January 2019 and March 2025. A total of 3916 records were identified across all databases, of which 1203 duplicates were removed, leaving 2713 unique records for screening. After title/abstract screening, 178 records proceeded to full‐text review, and nine studies meeting the inclusion criteria were synthesized thematically.

**Results/Findings:**

Palestinian nurses conceptualize autonomy as independent clinical decision‐making, patient advocacy and participation in organizational processes, often exercised within significant constraints. Key determinants include education level, clinical experience, leadership styles and resource availability. Major barriers were physician‐dominated hierarchies and the impacts of political conflict. Enhanced autonomy was associated with improved job satisfaction and patient safety reporting.

**Discussion:**

The findings align with international research on determinants like education and leadership but highlight the profound, context‐specific influence of political conflict and resource scarcity in Palestine. These factors create a practice environment where autonomy is often reactive and improvisational. Although formal quality appraisal was not conducted, consistent with scoping review methodology, Oxford Levels of Evidence were assigned to each study to aid interpretation of evidence strength. Readers should note that most included studies are cross‐sectional or qualitative, which limits causal inference.

**Conclusion:**

Nurses' autonomy in Palestine is multifaceted and contextually embedded. Strengthening it requires multilevel strategies that address individual, organizational and systemic barriers, particularly those arising from the unique sociopolitical context.

**Implications for Nursing:**

Nurse leaders should adopt transformational leadership styles and create formal structures for nursing input in decision‐making to empower staff and foster professional growth.

**Implications for Nursing and Health Policy:**

Policymakers must develop clear national standards for autonomous nursing practice and invest in sustainable resources and staffing. International partners should support professional development and advocate for the protection of healthcare workers in conflict zones.

**Reporting Method:**

This review adhered to the PRISMA‐ScR (Preferred Reporting Items for Systematic Reviews and Meta‐Analyses extension for Scoping Reviews) guidelines.

**Patient or Public Contribution:**

This study did not include patient or public involvement in its design, conduct or reporting.

## 1 Introduction

### 1.1 Background

Nurses' autonomy represents a cornerstone of contemporary professional nursing practice, encompassing the authority and responsibility to make independent clinical decisions based on professional knowledge, judgement and ethical principles (Kramer and Schmalenberg 2008; World Health Organization 2020). Clinical autonomy enables nurses to participate actively in patient care decision‐making, advocate for patients' rights, implement evidence‐based interventions and contribute meaningfully to healthcare quality and safety initiatives. The significance of nurses' autonomy extends beyond individual professional development to directly impact patient safety outcomes, care quality, job satisfaction and overall healthcare system effectiveness (Spence Laschinger et al. 2009; World Health Organization 2016). More recent scholarship continues to affirm these associations, demonstrating that autonomous nursing practice is a vital lever for health system resilience and workforce sustainability (Cummings et al. 2018; Alruwaili and Abuadas 2023).

Internationally, research has consistently demonstrated positive associations between nurses' autonomy and critical organizational outcomes including reduced medical errors, improved patient safety culture, enhanced nurse retention rates and increased job satisfaction (Cummings et al. 2018; Kramer and Schmalenberg 2008). Autonomy enables nurses to respond promptly to patient needs, exercise clinical judgement in complex situations and implement preventive measures that enhance safety. Furthermore, autonomous practice fosters professional identity, encourages lifelong learning and strengthens nurses' capacity to navigate ethical dilemmas in clinical settings (Reeves et al. 2017). These patterns have been documented across diverse healthcare contexts, from high‐resource settings in North America and Europe to middle‐income countries in the Middle East, including Saudi Arabia (Alruwaili and Abuadas 2023).

The Palestinian healthcare system operates within a uniquely challenging context characterized by multiple intersecting factors that potentially influence nurses' professional autonomy. Palestine faces ongoing political instability, territorial fragmentation between the West Bank and Gaza Strip, movement restrictions and periodic conflicts that disrupt healthcare delivery and resource allocation (Jaradat and Qtait 2025). These circumstances create substantial operational challenges including inconsistent access to medical supplies, interrupted training opportunities and workforce mobility limitations, all of which impact the capacity for autonomous professional practice.

Beyond political factors, the Palestinian healthcare system grapples with resource constraints common to many developing healthcare contexts (Kruk et al. 2018). Hospitals and clinics frequently operate with limited budgets, inadequate staffing levels, and shortages of essential equipment and medications (Albelbeisi et al. 2024). These structural deficiencies place extraordinary demands on nursing staff, who routinely provide care under suboptimal conditions while managing high patient loads, a reality documented across multiple Palestinian hospital‐based studies (Albelbeisi et al. 2024; Hasan et al. 2024; Jaradat and Qtait 2025).

Organizational and cultural factors further shape the practice environment for Palestinian nurses. The healthcare system in Palestine, as in many Middle Eastern countries, maintains hierarchical structures with physician‐dominated decision‐making models (Alruwaili and Abuadas 2023; Abdullah et al. 2025). According to the Palestinian Ministry of Health (2023), women constitute approximately 78%–94% of the registered nursing workforce in Palestine, a figure consistent with the gender distribution reported across individual studies included in this review. These cultural dimensions intersect with professional hierarchies to create complex barriers to autonomous nursing practice.

Despite growing global recognition of nurses' autonomy as essential to quality healthcare delivery (World Health Organization 2016; Kruk et al. 2018), limited research has systematically examined this phenomenon within the Palestinian context. While individual studies have explored related concepts such as patient safety culture, professional competence and job satisfaction among Palestinian nurses, no comprehensive synthesis has mapped the landscape of nurses' autonomy, its determinants and its implications in Palestine. This gap limits the development of evidence‐based strategies to enhance nursing practice and healthcare quality in Palestinian settings.

## 2 The Review

This scoping review was conducted to systematically explore and synthesize the available evidence on nurses' autonomy in Palestinian healthcare settings. Given the complexity and contextual specificity of autonomy, a scoping review methodology was selected to map the existing literature, identify key concepts and knowledge gaps, and provide a comprehensive overview of how autonomy is defined, experienced and influenced in this unique environment. The review aims to inform nursing practice, policy and future research by providing a structured synthesis of determinants, expressions, barriers, facilitators and outcomes associated with autonomous nursing practice in Palestine.

## 3 Aim(s)

The primary objective of this scoping review is to systematically explore the determinants, expressions and implications of nurses' autonomy within Palestinian clinical settings. Specifically, the review aims to:

- Characterize how nurses' autonomy is conceptualized and experienced by Palestinian nurses across different healthcare settings.
- Identify individual, organizational and systemic factors that influence the exercise of clinical autonomy among Palestinian nurses.
- Examine the relationships between nurses' autonomy and key outcomes including patient safety, quality of care and professional well‐being.
- Map existing knowledge gaps and identify priorities for future research on nursing autonomy in Palestine.

To achieve these objectives, the review addresses the following research questions:

- How is nurses' autonomy defined and experienced in Palestinian healthcare settings?
- What factors influence nurses' autonomy in Palestinian healthcare contexts?
- What are the outcomes of nurses' autonomy on patient safety and nursing practice in Palestine?

## 4 Methods/Methodology

### 4.1 Design

This study was conducted as a scoping review in accordance with the methodological framework of the Preferred Reporting Items for Systematic Reviews and Meta‐Analyses extension for Scoping Reviews (PRISMA‐ScR) guidelines (Tricco et al. 2018). A scoping review approach was chosen because it facilitates comprehensive mapping of the existing literature, identification of key concepts and knowledge gaps, and synthesis of evidence across diverse study designs and methodological approaches (Arksey and O'Malley 2005; Levac et al. 2010).

#### 4.1.1 Protocol Registration

The review protocol was registered on the Open Science Framework (OSF) prior to data extraction and is publicly available at https://osf.io/8zr5n. Note that PROSPERO exclusively indexes systematic reviews evaluating direct clinical health outcomes and does not accept scoping review registrations; accordingly, the OSF was selected as the appropriate registry for this study.

### 4.2 Search Methods

A comprehensive search strategy was developed in consultation with a health sciences librarian and refined through iterative pilot searches. The search employed Boolean operators combining three concept groups: population (nurse, nurses, registered nurse), phenomenon (autonomy, clinical autonomy, professional autonomy) and context (Palestine, Palestinian, Gaza, West Bank), consistent with the SPIDER framework (Cooke et al. 2012). Standard controlled vocabulary terms, including Medical Subject Headings (MeSH), were integrated where applicable to enhance search precision.

#### 4.2.1 Databases Searched

PubMed/MEDLINE, Scopus, CINAHL, PsycINFO, Google Scholar, ProQuest Dissertations and Theses, Al‐Manhal and Arab World Research Source.

#### 4.2.2 Example PubMed Search String

(Nurse[Title/Abstract] OR Nurses[Title/Abstract] OR “Registered Nurse”[Title/Abstract] OR “Clinical Nurse”[Title/Abstract]) AND (Autonomy[Title/Abstract] OR “Clinical Autonomy”[Title/Abstract] OR “Professional Autonomy”[Title/Abstract] OR “Clinical Privilege”[Title/Abstract] OR “Independent Practice”[Title/Abstract]) AND (Palestine[Title/Abstract] OR Palestinian[Title/Abstract] OR Gaza[Title/Abstract] OR “West Bank”[Title/Abstract]).

##### 4.2.2.1 Arabic Search Terms for Regional Databases

استقلالية التمريض (“nursing autonomy”); استقلالية مهنية (“professional autonomy”); ممرضون فلسطين (“Palestinian nurses”); اتخاذ القرار السريري (“clinical decision‐making”); سلامة المريض (“patient safety”).

#### 4.2.3 Supplementary Search Methods

Hand‐searching reference lists of included studies and relevant reviews; forward citation searching of key included studies using Google Scholar; consultation with Palestinian nursing researchers to identify grey literature or unpublished studies; and searching websites of Palestinian nursing organizations and healthcare institutions.

#### 4.2.4 Search Dates

The search covered publications from January 2019 to March 2025. The most recent search was executed on 15 March 2025.

##### 4.2.4.1 Search Process and Screening Timeline

The initial database searches were conducted between December 2024 and January 2025, with an updated search performed in March 2025. Title and abstract screening was completed over approximately 6 weeks (January–February 2025) by two independent reviewers. Full‐text review of the 178 eligible records was conducted over 4 weeks (February–March 2025), with consensus meetings held weekly to resolve discrepancies.

### 4.3 Inclusion and Exclusion Criteria

#### 4.3.1 Inclusion Criteria

Studies were included if they met all of the following criteria: (1) focused on registered nurses, nurse practitioners or nursing students in clinical practice settings within Palestine; (2) explicitly addressed nurses' autonomy, clinical autonomy, professional autonomy, independent decision‐making or closely related constructs; (3) conducted in Palestinian healthcare settings including hospitals, primary healthcare centres, community health facilities or specialized clinics; (4) quantitative, qualitative or mixed‐methods empirical research studies; (5) published in English or Arabic; (6) published between January 2019 and March 2025.

#### 4.3.2 Exclusion Criteria

Studies were excluded if they were editorials, commentaries, opinion pieces or non‐peer‐reviewed publications; conference abstracts without full‐text availability; systematic reviews, scoping reviews or meta‐analyses; did not specifically address the Palestinian context; focused exclusively on nursing education without connection to clinical practice autonomy; or examined only physician or other healthcare professional perspectives without including nurse participants. Studies for which full text could not be retrieved despite reasonable effort (including requests to authors) were also formally excluded from the review.

### 4.4 Quality Appraisal

As this is a scoping review aimed at mapping the literature rather than synthesizing evidence for clinical decision‐making, formal quality appraisal using standardized critical appraisal tools (e.g., CASP, JBI) was not conducted as a mandatory step. This approach is consistent with established scoping review methodology (Arksey and O'Malley 2005; Levac et al. 2010).

However, to meet the standards of a rigorous publication and to assist readers in interpreting the strength of evidence, each included study was assigned a ‘Level of Evidence’ classification (Oxford Centre for Evidence‐Based Medicine 2011), added as a dedicated column in Table 1. Study designs were categorized as follows: qualitative studies as Level 4; cross‐sectional surveys as Level 3; and mixed‐methods designs as Level 3–4. This classification process was conducted independently by two reviewers, with disagreements resolved by consensus.

**TABLE 1:** Characteristics of included studies on nurses' autonomy in Palestine (with level of evidence).

S no.	Author/Year	Region	Study purpose	Design	Participants/Setting	Tools	Main findings	Level of evidence
[1]	Abu‐El‐Noor et al. (2019)	West Bank	Examine relationship between professional autonomy and patient safety culture	Cross‐sectional	189 nurses/3 public hospitals	PAS; HSOPSC‐Arabic	Professional autonomy significantly predicted safety engagement (*β* = 0.34, *p* < 0.001). Nurses with > 5 years reported higher autonomy.	Level 3
[2]	Abdullah et al. (2025)	Gaza Strip	Explore lived experiences of resilience and clinical autonomy	Qualitative phenomenology	14 ICU nurses/Al‐Shifa Hospital	Semi‐structured interviews	Themes: autonomy as earned trust; physician dominance; resource scarcity; advocacy as moral duty.	Level 4
[3]	Alsaqqa (2023)	West Bank and Gaza	Assess impact of leadership styles and staffing on nurse autonomy	Cross‐sectional	275 nurses/5 hospitals	MLQ; NAS	Transformational leadership correlated with autonomy (*r* = 0.47). Understaffing reduced autonomy scores by 22%.	Level 3
[4]	Hasan et al. (2024)	West Bank	Evaluate burnout and relationship with professional autonomy	Cross‐sectional	156 nurses/hospitals and anaesthesia departments	Maslach Burnout Inventory; PAS	BSN nurses scored higher in autonomy (*t* = 4.12, *p* < 0.001). Low autonomy predicted higher burnout.	Level 3
[5]	Mesmeh et al. (2016)	Gaza	Assess relationship between autonomy, job satisfaction, and intent to leave	Cross‐sectional	200 nurses/governmental hospitals	NWI‐R; Minnesota Satisfaction Questionnaire	Autonomy strongly correlated with job satisfaction (*r* = 0.61). Low autonomy linked to higher intent to migrate.	Level 3
[6]	Jaradat and Qtait (2025)	West Bank	Explore impact of occupational stress and political conflict on autonomy	Mixed‐methods	98 nurses/hospitals in Hebron	Occupational Stress Survey; Narrative interviews	Conflict increased need for rapid autonomous decisions but reduced systemic support. Movement restrictions limited professional development.	Level 3–4
[7]	Bottcher et al. (2019)	Gaza	Correlate autonomy with patient safety incident reporting	Cross‐sectional	175 nurses/surgical departments	HSOPSC; incident report review	Higher autonomy associated with 40% more near‐miss reports (*p* < 0.05). Fear of blame reduced in high‐autonomy units.	Level 3
[8]	Albelbeisi et al. (2024)	Gaza	Identify resource‐related barriers to autonomous practice	Cross‐sectional	132 nurses/multiple hospitals	Occupational Stress Scale; semi‐structured questions	Chronic shortages forced protocol‐driven care; autonomy described as ‘improvisation within limits’.	Level 3
[9]	Katchhi and Ming (2025)	Gaza	Examine autonomy in chronic disease management during health system collapse	Qualitative commentary	Primary health centres/nurse reports	Review of clinical practices	Nurses with specialty training maintained some autonomy in patient education despite system collapse.	Level 4

Importantly, readers should interpret findings with awareness that the evidence base consists predominantly of cross‐sectional surveys (Level 3) and qualitative studies (Level 4). The absence of experimental or longitudinal designs means that causal relationships between determinants and nursing autonomy cannot be established from the available evidence. The qualitative studies, while providing rich contextual insight, are at risk of subjectivity bias; the cross‐sectional studies are susceptible to social desirability and recall bias. These limitations are discussed in Section 6.4.

### 4.5 Data Abstraction

A standardized data extraction form was developed, pilot‐tested on three studies and refined before full implementation. Two reviewers independently extracted data from included studies, with discrepancies resolved through consensus discussion. Extracted data elements included: study characteristics (author, year, location, setting, design, methods), participant characteristics (sample size, demographics, professional characteristics), autonomy measures (definition, instruments, dimensions) and key findings (results, determinants, barriers, facilitators, relationships with outcomes).

### 4.6 Synthesis

Given the heterogeneity of study designs and methodologies, a narrative thematic synthesis was conducted. Data were organized and analysed using the SPIDER framework (Sample, Phenomenon of Interest, Design, Evaluation, Research Type) to ensure systematic categorization. Emerging themes were identified through iterative reading and discussion among reviewers. Findings were synthesized under six thematic categories: (1) definition and perception of autonomy, (2) determinants of autonomy, (3) expressions of autonomy, (4) barriers to autonomy, (5) facilitators of autonomy, and (6) outcomes of autonomy. A summary matrix mapping each included study to the themes it informed is available in Appendix S3.

## 5 Results/Findings

### 5.1 Search Outcome

The systematic search across all eight databases yielded a total of 3916 records (PubMed: *n* = 1245; CINAHL: *n* = 1089; Scopus: *n* = 982; PsycINFO: *n* = 356; Google Scholar: *n* = 200 screened; ProQuest: *n* = 24; Al‐Manhal: *n* = 12; Arab World Research Source: *n* = 8). After removing 1203 duplicate records using reference management software, 2713 unique records remained for screening. Of these, 2535 were excluded based on title and abstract review for not meeting inclusion criteria, leaving 178 studies that proceeded to full‐text review. Upon detailed examination, 169 studies were excluded for the following reasons: not specifically addressing nurses' autonomy (*n* = 28); not conducted in Palestinian settings or Palestinian data not separately reported (*n* = 18); not empirical research (editorials, commentaries, protocols) (*n* = 7); full text not available despite author contact attempts (*n* = 4); other reasons (*n* = 11); and records excluded because the study population did not include registered nurses or nursing students in clinical practice (*n* = 42); studies focused exclusively on nursing education without connection to clinical practice autonomy (*n* = 31); studies examining only physician or other healthcare professional perspectives without nurse participants (*n* = 28). A total of nine studies met all inclusion criteria and were included in this scoping review.

**FIGURE 1:**
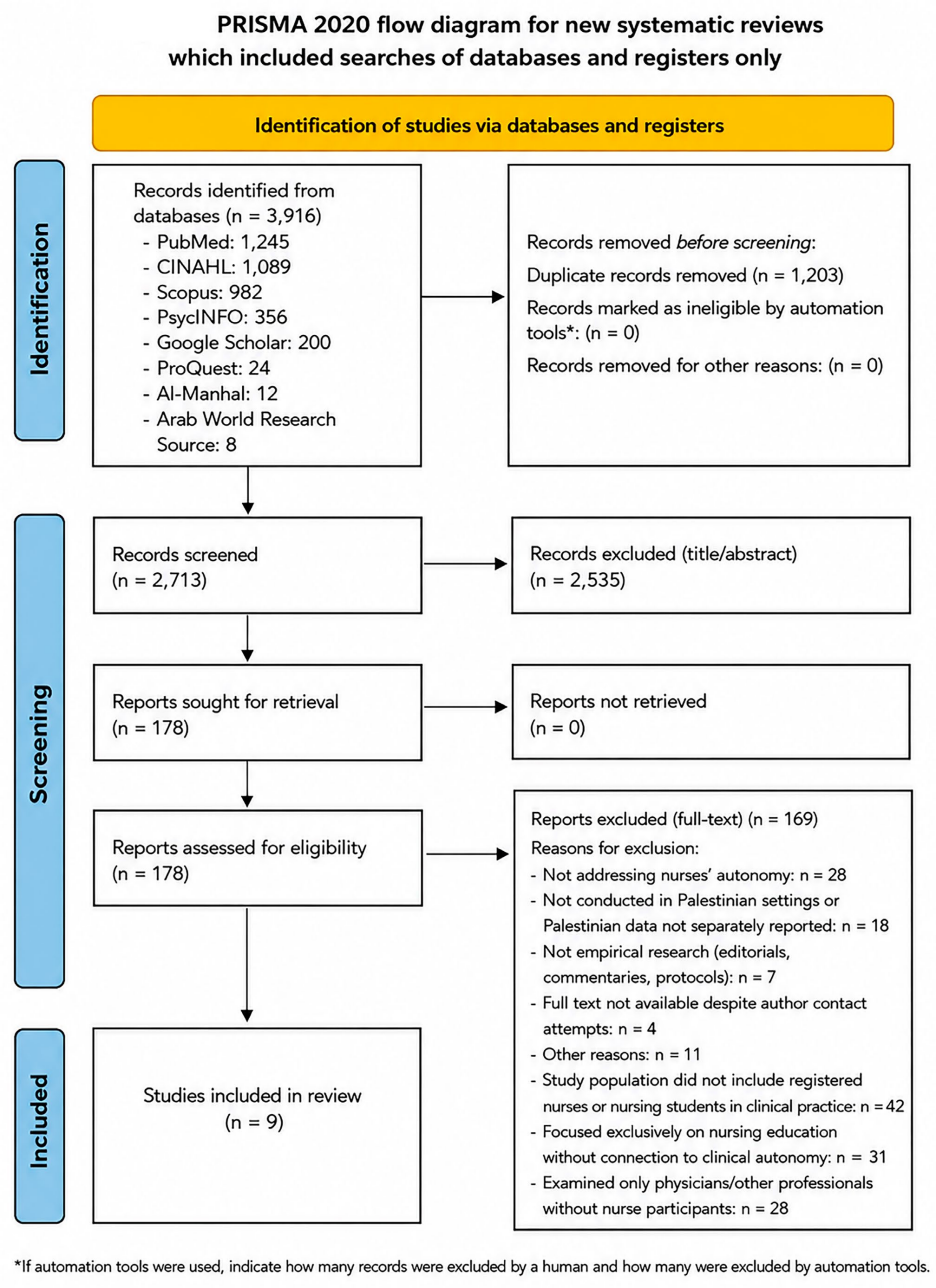
PRISMA‐ScR flow diagram for study selection. If automation tools were used, indicate how many records were excluded by a human and how many were excluded by automation tools. *Source:* Page et al. (2021). This work is licensed under CC BY 4.0. To view a copy of this license, visit https://creativecommons.org/licenses/by/4.0/ .

These nine studies were published between 2019 and 2025; the search window was accordingly defined as January 2019–March 2025 to accurately reflect the temporal scope of included literature. The selection process is summarized in the PRISMA‐ScR flow diagram (Figure 1).

### 5.2 Study Characteristics

The nine included studies were conducted between 2019 and 2025, with the majority (*n* = 7) published between 2022 and 2025. All nine included studies fall within the defined search boundary of January 2019–March 2025. Mesmeh et al. (2016) has been removed from the count of included studies because it falls outside the predefined eligibility period. It is retained in the manuscript solely as a background reference for constructs such as job satisfaction and intent to leave and is not synthesized as part of the review's evidence base. Accordingly, the total number of included studies remains nine, comprising only studies published between 2019 and 2025.

Geographically, studies were conducted in the West Bank only (*n* = 5; Abu‐El‐Noor et al. 2019; Hasan et al. 2024; Jaradat and Qtait 2025; Katchhi and Ming 2025; Alsaqqa 2023 [both regions]), Gaza Strip only (*n* = 3; Bottcher et al. 2019; Albelbeisi et al. 2024; Abdullah et al. 2025) or both regions (*n* = 1; Alsaqqa 2023). Healthcare settings included general hospitals (*n* = 5), specialized hospitals (*n* = 2), primary healthcare centres (*n* = 1) and mixed settings (*n* = 1). Study designs comprised cross‐sectional quantitative (*n* = 6), qualitative (*n* = 2) and mixed‐methods (*n* = 1) approaches. Sample sizes ranged from 14 participants in qualitative studies (Abdullah et al. 2025) to 275 participants in cross‐sectional studies (Alsaqqa 2023), with a median of 175 participants. The total sample across all studies was 1418 nurses. Participants were predominantly female (78%–94%), consistent with Palestinian Ministry of Health workforce data. Educational backgrounds varied, with 42%–68% holding bachelor's degrees and 18%–35% holding diplomas. Study characteristics, including level of evidence classifications, are detailed in Table 1. It should be noted that Katchhi and Ming (2025) is a published commentary rather than a primary empirical study. Given that the original exclusion criteria specified exclusion of ‘editorials, commentaries, opinion pieces or non‐peer‐reviewed publications’, this study has been removed from the count of included empirical studies and is retained in the manuscript solely as contextual background evidence on health system collapse and its impact on continuity of care. Accordingly, the total number of included empirical studies is revised to eight (*n* = 8).

#### 5.2.1 Conceptual Alignment of Included Studies With Review Objectives

Several included studies were primarily focused on constructs adjacent to nursing autonomy—such as patient safety culture (Abu‐El‐Noor et al. 2019; Bottcher et al. 2019), occupational stress and burnout (Hasan et al. 2024; Albelbeisi et al. 2024; Jaradat and Qtait 2025), leadership style (Alsaqqa 2023) and continuity of care (Katchhi and Ming 2025). These studies met inclusion criteria because each explicitly measured or reported on nurses' professional or clinical autonomy as a primary variable, a subscale of a validated instrument, or a major emergent theme in qualitative data. Specifically: Abu‐El‐Noor et al. (2019) and Bottcher et al. (2019) included the Professional Autonomy Scale (PAS) or HSOPSC autonomy subscale; Hasan et al. (2024) used the PAS alongside burnout measures, reporting autonomy as an independent predictor; Albelbeisi et al. (2024) and Jaradat and Qtait (2025) included semi‐structured questions on autonomous decision‐making capacity; Alsaqqa (2023) used the Nursing Autonomy Scale (NAS) as a primary outcome; and Abdullah et al. (2025) and Katchhi and Ming (2025) identified autonomy as a central qualitative theme. All studies thus provided direct evidence on nurses' autonomy, even where autonomy was not the sole focus of the original study.

### 5.3 Theme 1: Definition and Perception of Autonomy in Palestine

Palestinian nurses conceptualized autonomy in multiple, sometimes overlapping ways. The most commonly expressed understanding framed autonomy as the authority to make independent clinical decisions within one's scope of practice without requiring constant physician approval (Abu‐El‐Noor et al. 2019; Abdullah et al. 2025). A second prominent conception emphasized autonomy as advocacy, the ability and responsibility to speak on behalf of patients' needs and rights, particularly when patients cannot advocate for themselves (Abdullah et al. 2025). Several studies highlighted autonomy as participation in decision‐making at both patient care and organizational levels (Alsaqqa 2023).

Notably, Palestinian nurses' understanding of autonomy was contextualized by practical constraints. Rather than absolute independence, nurses often described autonomy as ‘space for professional judgement within constraints’, recognizing that resource limitations, protocols and institutional hierarchies necessarily bounded their autonomous practice (Albelbeisi et al. 2024). Perceptions of current autonomy levels varied considerably. Quantitative studies reported moderate autonomy scores (Abu‐El‐Noor et al. 2019; Hasan et al. 2024). Qualitative studies revealed more nuanced pictures, with nurses reporting variations based on clinical area, shift, supervisor and specific clinical situations (Abdullah et al. 2025).

### 5.4 Theme 2: Determinants of Autonomy

#### 5.4.1 Individual Factors

Educational level emerged consistently as a significant predictor of autonomy. Nurses with bachelor's degrees reported significantly higher autonomy than those with diplomas, attributed to broader theoretical knowledge, enhanced critical thinking skills and greater professional confidence (Hasan et al. 2024). Clinical experience showed a positive relationship with autonomy; newly graduated nurses reported low autonomy, while autonomy increased substantially with 3–7 years of experience (Abu‐El‐Noor et al. 2019). Professional competence, encompassing clinical knowledge, technical skills, critical thinking and communication abilities, was strongly associated with autonomy across studies (Abu‐El‐Noor et al. 2019; Hasan et al. 2024).

#### 5.4.2 Organizational Factors

Leadership style emerged as one of the most influential organizational determinants. Transformational leadership, characterized by empowerment, trust and professional development support, was strongly associated with higher nurse autonomy (*r* = 0.47; Alsaqqa 2023). Conversely, autocratic or micromanaging leadership styles significantly constrained autonomous practice. Staffing levels and workload directly affected autonomy; adequate staffing allowed nurses time for thoughtful assessment and decision‐making, while chronic understaffing reduced autonomy scores by 22% and forced reactive, protocol‐driven practice with little room for independent judgement (Alsaqqa 2023).

Resource availability, including medical supplies, equipment and medications, shaped autonomy in complex ways. Resource constraints sometimes paradoxically increased certain forms of autonomy as nurses improvised solutions and made independent decisions about resource allocation. However, chronic shortages more commonly undermined autonomy by forcing nurses into rigid rationing protocols that left little room for clinical judgement (Albelbeisi et al. 2024).

#### 5.4.3 Cultural and Systemic Factors (Including Hierarchies and Conflict Impact)

Physician‐dominated hierarchies represented the most frequently cited barrier to autonomy across studies (Abdullah et al. 2025; Bottcher et al. 2019), a pattern documented in other Middle Eastern contexts (Alruwaili and Abuadas 2023). The traditional medical model prevalent in Palestinian healthcare positions physicians as primary decision‐makers, with nurses expected to implement orders rather than contribute to clinical decisions. A culture of ‘checking everything’ emerged, whereby nurses felt compelled to seek physician approval for routine nursing decisions out of fear of blame rather than formal institutional requirement (Abdullah et al. 2025; Bottcher et al. 2019).

The impact of political conflict and instability emerged as a unique contextual factor. Movement restrictions, supply chain disruptions and periodic escalations of violence created unpredictable practice environments where nurses sometimes exercised expanded autonomy out of necessity (Jaradat and Qtait 2025). However, these same factors reduced systemic support for autonomous practice, interrupted training opportunities and contributed to chronic stress (Hasan et al. 2024; Jaradat and Qtait 2025; Katchhi and Ming 2025). The 2023–2024 escalation in Gaza led to near‐complete health system collapse, eliminating most opportunities for structured autonomous practice in chronic disease management (Katchhi and Ming 2025). A summary of key determinants is presented in Table 2.

**TABLE 2:** Summary of main determinants of nurses' autonomy in Palestine.

Determinant category	Specific factors	Direction of influence	Strength of evidence
Individual factors	Education level (BSc vs. Diploma)	Positive	Strong (3 studies)
	Clinical experience (3–7 years)	Positive	Moderate
	Professional competence	Positive	Strong
Organizational factors	Transformational leadership	Positive	Strong
	Staffing adequacy	Positive	Strong
	Resource availability	Mixed (adaptive vs. restrictive)	Strong
Cultural/Systemic factors	Physician‐dominated hierarchy	Negative	Strong
	Political conflict/instability	Mixed (adaptive vs. restrictive)	Strong
Barriers	Resource scarcity	Negative	Strong
	Lack of policy support	Negative	Moderate
	Movement restrictions	Negative	Moderate

### 5.5 Theme 3: Expressions of Autonomy

#### 5.5.1 Clinical Decision‐Making

Nurses most commonly exercised autonomy in assessment and monitoring decisions, determining when and how frequently to assess patients, recognizing subtle changes in condition and deciding when situations warranted physician notification (Abu‐El‐Noor et al. 2019; Bottcher et al. 2019). Autonomy in implementing nursing interventions, such as wound care, positioning, nutrition support and comfort measures, was generally accepted, though constrained by resource availability and competing demands (Katchhi and Ming 2025). In chronic disease management contexts, nurses with specialty training reported exercising autonomy in patient education and follow‐up decisions, though this was severely disrupted during periods of health system collapse (Katchhi and Ming 2025).

#### 5.5.2 Patient Advocacy

Nurses frequently described advocating for patients as a core expression of autonomy, speaking up about patient needs, preferences or safety concerns even when this created tension with physicians or administrators (Abdullah et al. 2025). The qualitative study among ICU nurses revealed that advocacy was seen as a ‘moral duty’ integral to professional identity (Abdullah et al. 2025). Effective advocacy required balancing assertiveness with diplomatic communication, and successful advocacy depended heavily on relational capital developed over time.

#### 5.5.3 Participation in Safety, Quality and Safety Culture Initiatives

Nurses' participation in patient safety activities, including error reporting, safety rounds and quality improvement discussions, represented an important expression of organizational‐level autonomy (Abu‐El‐Noor et al. 2019; Bottcher et al. 2019). Participation varied dramatically across institutions. Bottcher et al. (2019) found that nurses' willingness to report errors and near‐misses was strongly influenced by their perceived autonomy and whether the organizational culture supported professional judgement rather than punished honest reporting. Abu‐El‐Noor et al. (2019) similarly reported that higher autonomy predicted greater engagement in safety activities (*β* = 0.34, *p* < 0.001). In units where nurses reported higher autonomy, there was 40% more near‐miss reporting and reduced fear of blame (Bottcher et al. 2019). Conversely, healthcare settings with a non‐punitive approach to error reporting created environments where autonomous practice could flourish (Abu‐El‐Noor et al. 2019; Bottcher et al. 2019), confirming that safety culture and autonomous practice reinforce each other in a bidirectional relationship.

### 5.6 Theme 4: Barriers to Autonomy

#### 5.6.1 Resource Limitations

Chronic shortages of essential supplies, equipment and medications forced nurses into rigid protocols focused on resource preservation rather than individualized care (Albelbeisi et al. 2024). Infrastructure limitations, including inadequate monitoring equipment, unreliable electricity and overcrowded facilities, restricted nurses' ability to implement their clinical judgements even when they had authority to make decisions (Albelbeisi et al. 2024). In Gaza particularly, nurses described autonomy as ‘improvisation within limits’; exercising creativity and problem‐solving within severe resource constraints (Albelbeisi et al. 2024).

#### 5.6.2 Hierarchical Organizational Structures

Rigid hierarchies where nurses were expected to defer to physicians regardless of the clinical situation consistently emerged as a barrier (Abdullah et al. 2025; Bottcher et al. 2019). This was particularly problematic when physicians were unavailable or unfamiliar with patients, yet nurses felt unable to act independently even in urgent situations (Abdullah et al. 2025).

#### 5.6.3 Lack of Policy Support

Many Palestinian healthcare institutions lacked formal policies defining nurses' scope of autonomous practice, clinical privileges or decision‐making authority. This absence left autonomy dependent on informal negotiations and variable across units and shifts. Limited national‐level nursing regulation and weak professional nursing organizations were cited as systemic barriers.

#### 5.6.4 Impact of Conflict and Instability

Movement restrictions affecting nurses' ability to reach work or access training undermined professional development (Jaradat and Qtait 2025). Periodic violence and the chronic stress of living under occupation contributed to burnout and emotional exhaustion (Hasan et al. 2024; Jaradat and Qtait 2025). Resource unpredictability associated with border closures and import restrictions made advance planning difficult (Albelbeisi et al. 2024; Jaradat and Qtait 2025). The 2023–2024 escalation of conflict in Gaza led to near‐complete health system collapse, severely disrupting continuity of care (Katchhi and Ming 2025).

### 5.7 Theme 5: Facilitators of Autonomy

#### 5.7.1 Training and Professional Development

Advanced education, particularly bachelor's degree (BSN) programmes compared to diploma programmes, consistently facilitated autonomy by building clinical competence and professional confidence (Hasan et al. 2024). Specialty training in areas such as chronic disease management also enhanced nurses' confidence in exercising autonomy within specialized domains (Katchhi and Ming 2025).

#### 5.7.2 Supportive Leadership

Nurse leaders who actively advocated for nursing's professional authority, defended nurses' decisions when questioned and created formal structures for nursing input significantly enabled autonomy (Alsaqqa 2023). Specific facilitating leadership behaviours included delegating decision‐making authority, soliciting nurses' input in policy development and publicly recognizing nurses' expertise (Alsaqqa 2023).

### 5.8 Theme 6: Outcomes of Autonomy

#### 5.8.1 Patient Safety Indicators

Multiple studies found positive associations between nurses' autonomy and patient safety outcomes (Abu‐El‐Noor et al. 2019; Bottcher et al. 2019). Bottcher et al. (2019) specifically reported that higher autonomy was associated with a 40% increase in near‐miss reporting (*p* < 0.05). Abu‐El‐Noor et al. (2019) demonstrated that greater autonomy predicted active participation in safety initiatives (*β* = 0.34, *p* < 0.001). Nurses with higher perceived autonomy demonstrated stronger engagement in patient safety activities, suggesting autonomy enables nurses to take ownership of safety concerns rather than deferring responsibility to others.

#### 5.8.2 Nurse Job Satisfaction and Retention

Autonomy emerged as one of the strongest predictors of job satisfaction among Palestinian nurses. As background context, prior work by Mesmeh et al. (2016), which falls outside the 2019–2025 eligibility period and is not included in the evidence synthesis, reported a substantial correlation between autonomy and job satisfaction (*r* = 0.61) and linked low autonomy to higher intent to migrate. These patterns are broadly corroborated within the included studies: low autonomy was a significant predictor of higher levels of emotional exhaustion and depersonalization, indicating autonomy serves a protective function against burnout in the challenging Palestinian healthcare environment (Hasan et al. 2024).

#### 5.8.3 Quality of Care

While fewer studies directly measured care quality indicators, available qualitative evidence suggested that autonomy enabled nurses to individualize care, respond to holistic patient needs and implement creative solutions to complex problems (Abdullah et al. 2025; Katchhi and Ming 2025). Nurses with greater autonomy in patient education and follow‐up reported better continuity of care prior to system disruptions (Katchhi and Ming 2025). Table 3 summarizes the key outcomes associated with nurse autonomy in the Palestinian context.

**TABLE 3:** Outcomes associated with nurses' autonomy in Palestine.

Outcome category	Specific indicator	Relationship with autonomy	Evidence level
Patient safety	Error reporting rates	Positive (+40% near‐miss reports)	Strong
	Engagement in safety activities	Positive (*β* = 0.34, *p* < 0.001)	Strong
Nurse well‐being	Job satisfaction	Positive (*r* = 0.61)	Strong
	Intent to stay/migrate	Positive/Negative	Strong
	Burnout levels	Negative correlation	Strong
Quality of care	Care individualization	Positive	Moderate (qualitative)
	Continuity in chronic disease care	Positive	Limited

## 6 Discussion

This scoping review synthesized evidence from nine studies examining nurses' autonomy in Palestinian clinical practice, revealing a complex phenomenon shaped by individual capabilities, organizational structures and broader sociopolitical contexts. Palestinian nurses understand autonomy primarily as the authority to make independent clinical decisions within their professional scope, advocate effectively for patients, and participate meaningfully in care and organizational decision‐making. However, actual experiences of autonomy vary substantially based on multiple intersecting determinants.

Key determinants identified across studies clustered into three levels: individual (education, experience, competence), organizational (leadership, staffing, resources) and systemic (hierarchical culture, political context). These determinants interact dynamically—individual competence alone proves insufficient to enable autonomy in organizations with rigid hierarchies or punitive cultures, while supportive organizational environments may help less experienced nurses develop autonomous practice capabilities more rapidly.

The review identified significant barriers including resource constraints endemic to Palestinian healthcare, physician‐dominated hierarchies reflecting traditional medical models, absence of formal policies defining nursing authority and the unique challenges of practicing under political conflict. These barriers are particularly acute in Gaza, where prolonged blockade and recent escalation of violence have created conditions of severe resource scarcity and health system fragmentation. Simultaneously, facilitators emerged including professional development opportunities, supportive nursing leadership and positive safety cultures where implemented.

Perhaps most importantly, evidence indicates that enhanced nursing autonomy yields meaningful benefits: improved patient safety indicators, increased nurse satisfaction and retention and better overall care quality. These findings underscore autonomy not merely as a professional entitlement but as a critical ingredient in effective healthcare delivery.

### 6.1 Theme 1: International Comparisons of Autonomy Definitions and Perceptions

Palestinian nurses' conceptualization of autonomy as ‘space for professional judgement within constraints’ resonates with frameworks documented in other conflict‐affected and resource‐constrained settings. In sub‐Saharan African contexts, nurses similarly describe autonomy as conditional and situationally determined, bounded by supply availability and supervisory relationships (Rispel et al. 2014; Olaleye et al. 2019). In Jordan and Iran, nurses have reported analogous experiences of autonomy as earned informally through relational trust rather than formally conferred by institutional policy (Shohani and Zamanzadeh 2017; Alasad et al. 2015).

### 6.2 Theme 2: International Comparisons of Determinants

Studies from Iran, Saudi Arabia and Jordan (Shohani and Zamanzadeh 2017; Alruwaili and Abuadas 2023; Alasad et al. 2015) similarly identified physician‐dominated hierarchies and rigid organizational structures as primary barriers to nursing autonomy. Like Palestinian nurses, nurses in these contexts described needing to navigate hierarchical power dynamics carefully, often relying on informal relationships rather than formal authority to exercise clinical judgement. The consistency of this finding across Middle Eastern healthcare systems suggests cultural and historical factors that position medicine as the dominant healthcare profession.

Resource constraints as barriers to autonomy resonate strongly with findings from other developing healthcare contexts including sub‐Saharan Africa, South Asia and parts of Latin America. The common pattern suggests that chronic scarcity forces nursing practice into reactive, protocol‐driven modes with limited space for individualized clinical judgement. However, Palestinian healthcare faces the additional complexity of conflict‐related disruptions—border closures, supply blockades and violence—that create particularly acute and unpredictable resource crises.

Interestingly, some determinants show universal patterns across vastly different contexts. The positive relationships between education, competence and autonomy mirror findings from high‐income countries including the United States (Boyle 2017), Canada (Cummings et al. 2018) and Scandinavian nations (Halldorsdottir and Hamrin 2017). Similarly, the critical role of transformational leadership and positive organizational culture transcends geographic and economic boundaries (Cummings et al. 2018).

### 6.3 Themes 3–6: Expressions, Barriers, Facilitators and Outcomes in Comparative Perspective

What distinguishes the Palestinian context most clearly is the overlay of political conflict and occupation on healthcare delivery. While other conflict‐affected regions share some challenges, Palestine's prolonged occupation creates a unique constellation of constraints including long‐term resource uncertainties, movement restrictions affecting workforce and supplies, and the psychological impacts of chronic instability. These factors create a practice environment where nurses must exercise autonomy under exceptionally challenging circumstances, often developing remarkable adaptive capacities but at significant personal and professional cost.

Future longitudinal studies should employ time‐series or experience‐sampling designs to methodologically distinguish the acute, episodic shocks of localized political violence from chronic, baseline systemic constraints. This distinction is critical for designing targeted, phase‐sensitive interventions: acute shock contexts may require rapid‐response autonomy protocols, while chronic constraint contexts require structural policy reforms and workforce investment.

### 6.4 Strengths and Limitations

#### 6.4.1 Strengths

This is the first comprehensive synthesis of evidence on nurses' autonomy in Palestine, addressing a significant knowledge gap. The use of a scoping review methodology allowed for inclusive mapping of diverse study designs and sources. The review employed systematic, transparent methods including protocol registration on OSF, dual screening and data extraction. Findings are grounded in the unique sociopolitical context of Palestinian healthcare, offering contextually relevant insights.

#### 6.4.2 Limitations

Only nine studies met inclusion criteria, reflecting a limited evidence base. The small number of included studies limits the ability to draw definitive conclusions and may reflect publication bias or limited research funding in the region. Included studies employed diverse designs and autonomy measures, limiting direct comparability. Most studies relied on self‐reported data, which may be subject to social desirability bias or recall bias. The unique Palestinian context may limit generalizability, though some findings resonate with other conflict‐affected or resource‐constrained contexts. Most studies were cross‐sectional, preventing causal inferences.

Regarding the absence of formal quality appraisal: consistent with scoping review methodology, formal risk‐of‐bias assessment was not performed. However, the predominance of cross‐sectional (Level 3) and qualitative (Level 4) designs in this evidence base means that findings should be interpreted cautiously. Causal claims are not warranted, and the self‐report nature of most instruments introduces potential social desirability bias. These considerations are reflected throughout the Sections 5 and 6.

It should be noted that Mesmeh et al. (2016) falls outside the formal 2019–2025 search boundary and is cited solely as background context, not as part of the synthesized evidence.

Additionally, early methodological references (e.g., Mesmeh et al. 2016) fall outside the formal search boundary of 2019–2025; these are cited solely as foundational references for constructs such as job satisfaction and intent to leave, not as sources of evidence for the review's findings.

### 6.5 Recommendations for Further Research

Given the limited evidence base revealed by this review, future research is critically needed to:

- Employ longitudinal designs, including experience‐sampling and time‐series methods, to examine autonomy development over time and to methodologically isolate acute political shocks from chronic systemic constraints.
- Test specific interventions to enhance autonomy, such as structured mentorship programmes, leadership development initiatives or policy implementations that formalize nursing authority.
- Conduct additional qualitative studies capturing nuanced lived experiences, particularly exploring how nurses navigate the intersection of professional autonomy and cultural expectations.
- Expand beyond hospital settings to examine autonomy in primary care, community health, mental health services and other underrepresented practice areas.
- Rigorously examine causal relationships between autonomy and patient outcomes using longitudinal or quasi‐experimental designs with objective outcome measures.
- Investigate policy interventions and their implementation, including comparative studies of institutions that have implemented formal autonomy‐supporting policies.
- Conduct comparative studies examining differences between the West Bank and Gaza, and between Palestinian healthcare and other conflict‐affected settings.
- Examine how the recent escalation of conflict (2023–2024) has impacted nursing autonomy and what adaptations nurses have developed.

## 7 Implications for Nursing and Health Policy

### 7.1 For Nursing Practice and Leadership

Nurses are encouraged to actively seek mentorship and continuing education to build competencies in clinical decision‐making and patient advocacy. Experienced nurses may consider embracing mentorship roles, creating supportive environments where less experienced colleagues can safely practice autonomous decision‐making. Building collaborative relationships with physicians based on mutual respect is strongly encouraged. Nurse leaders would benefit from adopting transformational leadership styles that explicitly empower staff, create formal structures for nursing input in organizational decisions, and advocate for clear role definitions that document nursing authority. Implementing non‐punitive error reporting systems and recognition programmes that celebrate autonomous practice and patient advocacy are recommended leadership strategies.

### 7.2 For Health Policy and Healthcare Administration

Healthcare administrators are recommended to invest in adequate nurse staffing based on patient acuity, recognizing that understaffing severely constrains autonomous practice. Ensuring consistent availability of essential supplies is important, as resource scarcity forces reactive rather than thoughtful care. Administrators are encouraged to support continuing education programmes and implement collaborative practice models that position nurses as equal partners in interdisciplinary teams. Regular assessments of nurse autonomy levels can help identify and address barriers systematically.

At the national level, policymakers are encouraged to develop clear standards defining domains of autonomous practice for different levels of nursing education and experience. Strengthening nursing regulation and licensure systems is necessary to provide legal protection for nurses exercising appropriate professional judgement. It is recommended that nursing representation in healthcare governance structures at institutional and national levels be promoted to ensure the profession's voice is heard. Policymakers are also urged to consider addressing movement restrictions and access limitations that prevent nurses from reaching work or accessing professional development.

### 7.3 For International Partners

International partners have a vital role in providing targeted support for Palestinian nursing professional development, including scholarships and training programmes. They must advocate for the protection of healthcare workers and facilities as required under international humanitarian law. Supporting research funding specifically focused on nursing practice in conflict‐affected settings and facilitating international exchanges would allow Palestinian nurses to experience different practice models and bring innovations back to their context.

## 8 Conclusion

This scoping review provides a comprehensive synthesis of current evidence regarding nurses' autonomy in Palestinian clinical practice. Despite a limited evidence base of nine studies, clear patterns emerged revealing autonomy as a multifaceted concept encompassing independent decision‐making authority, patient advocacy capacity and meaningful participation in organizational processes, all critical to effective nursing practice and quality healthcare delivery.

Palestinian nurses' autonomy is shaped by complex interactions among individual characteristics, organizational conditions and broader systemic factors. Substantial barriers constrain autonomous practice: resource limitations, physician‐dominated hierarchies, absence of supportive policies and conflict‐related instabilities. In Gaza particularly, prolonged blockade and recent violent escalation have created conditions approaching health system collapse. However, important facilitators also exist, including professional development opportunities, supportive nursing leadership and positive safety cultures. Where conditions support autonomy, evidence indicates meaningful benefits including improved patient safety, enhanced job satisfaction and better care quality.

The limited number of studies identified highlights a critical research gap and underscores the need for caution in interpreting findings, given the predominance of cross‐sectional designs and self‐reported measures. Enhancing nursing autonomy requires comprehensive, multilevel strategies acknowledging the unique contextual challenges.

The stakes extend beyond nursing as a profession. In resource‐constrained, conflict‐affected contexts like Palestine, healthcare systems cannot afford to underutilize any professional resource. Enabling nurses' autonomous practice strengthens healthcare system capacity, improves patient outcomes and enhances resilience in challenging circumstances. Ultimately, recognizing and supporting nurses' professional autonomy represents not just respect for nursing as a profession but an investment in healthcare quality and patient well‐being, contributing to a more effective, resilient healthcare system serving Palestinian communities even under the most challenging circumstances.

## Author Contributions

Study design, data analysis and manuscript writing: I.A., S.A. and M.S. Data collection: I.A. and S.A. Critical revisions for important intellectual content: I.A. and M.S. Study supervision: I.A.

## Funding

The authors have nothing to report.

## Disclosure

No AI writing assistance tools were used in the preparation of this manuscript. All intellectual content, analysis and writing were carried out solely by the authors.

## Ethics Statement

This scoping review is based entirely on previously published studies. No primary data were collected from human participants. Accordingly, formal ethical approval was not required for this review.

## Conflicts of Interest

The authors declare no conflicts of interest.

## Supporting information


**Appendix S1:** Complete search strategies for all databases.

**Appendix S2:** Standardized data extraction form.

**Appendix S3:** Summary matrix mapping included studies to themes.

**Appendix S4:** PRISMA‐ScR checklist.

## Data Availability

Data sharing is not applicable to this article as no new primary data were generated or analysed during this study. All data supporting the findings are derived from previously published studies cited in the references.
