# Supplementary material for: Exploring Professional Autonomy Among Palestinian Nurses: A Comprehensive Scoping Review of Determinants, Barriers and Clinical Practice Implications
Source: Nurs Open. 2026 Jun 17;13(6):e70652. doi: 10.1002/nop2.70652 (PMC13275553; doi:10.1002/nop2.70652)
Supplement: Supplementary file 1 — Appendix S1: Complete search strategies for all databases. [file NOP2-13-e70652-s002.docx]

**COMPLETE SEARCH STRATEGIES FOR ALL DATABASES**

Review: Exploring Professional Autonomy Among Palestinian Nurses: A Scoping Review

Date of search: 15 March 2025

-------------------------------------------------------------------------------

1. PubMed/MEDLINE (search date: 15 March 2025)

Search string:

("Nurses"[Mesh] OR "Nursing Staff"[Mesh] OR "Nurse"[tiab] OR "Nurses"[tiab] OR "Registered Nurse"[tiab] OR "Clinical Nurse"[tiab]) AND ("Professional Autonomy"[Mesh] OR "Autonomy"[tiab] OR "Clinical Autonomy"[tiab] OR "Professional Autonomy"[tiab] OR "Clinical Privilege"[tiab] OR "Independent Practice"[tiab]) AND ("Palestine"[Mesh] OR "Palestine"[tiab] OR "Palestinian"[tiab] OR "Gaza"[tiab] OR "Gaza Strip"[tiab] OR "West Bank"[tiab])

Limits: 2019-2025; English or Arabic

Results: 1,245 records

-------------------------------------------------------------------------------

2. CINAHL (EBSCOhost) (search date: 15 March 2025)

Search string:

(MH "Nurses+") OR (TI nurse* OR AB nurse*) OR (TI "registered nurse" OR AB "registered nurse") AND (MH "Professional Autonomy") OR (TI autonom* OR AB autonom*) OR (TI "clinical autonomy" OR AB "clinical autonomy") AND (MH "Palestine") OR (TI Palestine OR AB Palestine) OR (TI Palestinian OR AB Palestinian) OR (TI Gaza OR AB Gaza) OR (TI "West Bank" OR AB "West Bank")

Limits: 2019-2025; English or Arabic

Results: 1,089 records

-------------------------------------------------------------------------------

3. Scopus (search date: 15 March 2025)

Search string:

TITLE-ABS-KEY(nurse* OR "registered nurse" OR "clinical nurse") AND TITLE-ABS-KEY(autonom* OR "clinical autonomy" OR "professional autonomy" OR "independent practice") AND TITLE-ABS-KEY(palestine OR palestinian OR gaza OR "west bank")

Limits: 2019-2025; English or Arabic

Results: 982 records

-------------------------------------------------------------------------------

4. PsycINFO (search date: 15 March 2025)

Search string:

(nurse* OR "registered nurse" OR "clinical nurse").ti,ab. AND (autonom* OR "clinical autonomy" OR "professional autonomy").ti,ab. AND (palestine OR palestinian OR gaza OR "west bank").ti,ab.

Limits: 2019-2025; English or Arabic

Results: 356 records

-------------------------------------------------------------------------------

5. Google Scholar (search date: 15 March 2025)

Search string:

intitle:nurse|nurses intitle:autonomy|"clinical autonomy"|"professional autonomy" Palestine|Palestinian|Gaza|"West Bank"

Limits: 2019-2025

Results: First 200 records screened

-------------------------------------------------------------------------------

6. ProQuest Dissertations and Theses (search date: 15 March 2025)

Search string:

(nurse* OR "registered nurse") AND (autonom* OR "clinical autonomy") AND (Palestine OR Palestinian OR Gaza OR "West Bank")

Limits: 2019-2025; English or Arabic

Results: 24 records

-------------------------------------------------------------------------------

7. Al-Manhal (Arabic database) (search date: 15 March 2025)

Search string (Arabic):

استقلالية التمريض OR استقلالية مهنية AND (فلسطين OR الضفة الغربية OR قطاع غزة)

English translation:

("nursing autonomy" OR "professional autonomy") AND (Palestine OR "West Bank" OR "Gaza Strip")

Limits: 2019-2025; Arabic language

Results: 12 records

-------------------------------------------------------------------------------

8. Arab World Research Source (search date: 15 March 2025)

Search string (Arabic):

ممرضون فلسطين OR اتخاذ القرار السريري AND سلامة المريض

English translation:

("Palestinian nurses" OR "clinical decision-making") AND "patient safety"

Limits: 2019-2025; Arabic language

Results: 8 records

-------------------------------------------------------------------------------

Notes:

- All searches were conducted on 15 March 2025

- Duplicates were removed using reference management software

- A supplementary updated search was performed on 15 March 2025 prior to submission

- Arabic terms were validated by a native Arabic-speaking researcher
