# Supplementary material for: Exploring Professional Autonomy Among Palestinian Nurses: A Comprehensive Scoping Review of Determinants, Barriers and Clinical Practice Implications
Source: Nurs Open. 2026 Jun 17;13(6):e70652. doi: 10.1002/nop2.70652 (PMC13275553; doi:10.1002/nop2.70652)
Supplement: Supplementary file 2 — Appendix S2: Standardized data extraction form. [file NOP2-13-e70652-s001.docx]

**STANDARDIZED DATA EXTRACTION FORM**

Review: Exploring Professional Autonomy Among Palestinian Nurses: A Scoping Review

-------------------------------------------------------------------------------

EXTRACTOR NAME: ________________________

DATE OF EXTRACTION: ________________________

STUDY ID (Ref #): ________________________

-------------------------------------------------------------------------------

SECTION A: STUDY CHARACTERISTICS

A1. Author(s): ________________________

A2. Year of publication: ________________________

A3. Country/Region: ☐ West Bank ☐ Gaza Strip ☐ Both ☐ Other: ______

A4. Healthcare setting: ☐ General hospital ☐ Specialized hospital ☐ Primary healthcare ☐ Community health ☐ Mixed ☐ Other: ______

A5. Study design: ☐ Cross-sectional ☐ Qualitative ☐ Mixed-methods ☐ Quasi-experimental ☐ RCT ☐ Other: ______

A6. Study purpose (aims): ________________________________________________

-------------------------------------------------------------------------------

SECTION B: PARTICIPANT CHARACTERISTICS

B1. Sample size (N): ________________________

B2. Participant type: ☐ Registered nurses ☐ Nurse practitioners ☐ Nursing students ☐ Other: ______

B3. Gender distribution (% female): ________________________

B4. Education level: ☐ Diploma (%) ___ ☐ Bachelor's (%) ___ ☐ Master's (%) ___ ☐ Doctorate (%) ___

B5. Mean years of experience: ________________________

B6. Clinical setting/department: ________________________________________________

-------------------------------------------------------------------------------

SECTION C: AUTONOMY MEASURES

C1. How was autonomy defined? (quote from study): ________________________

C2. Instrument/tool used (if quantitative):

☐ Professional Autonomy Scale (PAS)

☐ Nursing Autonomy Scale (NAS)

☐ NWI-R autonomy subscale

☐ Other: ________________________

C3. Dimensions of autonomy measured (check all that apply):

☐ Clinical decision-making

☐ Patient advocacy

☐ Participation in organizational processes

☐ Independent practice authority

☐ Other: ________________________

-------------------------------------------------------------------------------

SECTION D: KEY FINDINGS

D1. Main findings related to autonomy (themes or statistics):

________________________________________________

D2. Determinants identified (check all that apply):

☐ Education level ☐ Clinical experience ☐ Professional competence

☐ Leadership style ☐ Staffing adequacy ☐ Resource availability

☐ Physician-dominated hierarchy ☐ Political conflict

☐ Other: ________________________

D3. Barriers identified:

________________________________________________

D4. Facilitators identified:

________________________________________________

D5. Outcomes associated with autonomy (check all that apply):

☐ Patient safety ☐ Error reporting ☐ Job satisfaction

☐ Intent to stay/leave ☐ Burnout ☐ Quality of care

☐ Other: ________________________

-------------------------------------------------------------------------------

SECTION E: LEVEL OF EVIDENCE (Oxford CEBM 2011)

E1. Assigned Level of Evidence:

☐ Level 1 ☐ Level 2 ☐ Level 3 ☐ Level 4 ☐ Level 5

☐ Not applicable (qualitative: Level 4)

☐ Mixed-methods: Level ____

E2. Justification: ________________________________________________

-------------------------------------------------------------------------------

SECTION F: ADDITIONAL NOTES

F1. Any limitations noted by study authors:

________________________________________________

F2. Any conflicts of interest declared:

________________________________________________

F3. Reviewer notes/comments:

________________________________________________

-------------------------------------------------------------------------------

Form pilot-tested on 3 studies prior to full implementation.

Extraction performed independently by two reviewers; discrepancies resolved by consensus.
