# Supplementary material for: Exploring Professional Autonomy Among Palestinian Nurses: A Comprehensive Scoping Review of Determinants, Barriers and Clinical Practice Implications
Source: Nurs Open. 2026 Jun 17;13(6):e70652. doi: 10.1002/nop2.70652 (PMC13275553; doi:10.1002/nop2.70652)
Supplement: Supplementary file 3 — Appendix S3: Summary matrix mapping included studies to themes. [file NOP2-13-e70652-s003.docx]

**SUMMARY MATRIX MAPPING INCLUDED STUDIES TO THEMES**

Review: Exploring Professional Autonomy Among Palestinian Nurses: A Scoping Review

LEGEND:

✓ = Study informs this theme

✗ = Study does not inform this theme

| INCLUDED STUDY | THEME 1 Definition | THEME 2 Determinants | THEME 3 Expressions | THEME 4 Barriers | THEME 5 Facilitators | THEME 6 Outcomes |
| --- | --- | --- | --- | --- | --- | --- |
| 1. Abu-El-Noor et al. 2019 | ✓ | ✓ | ✓ | ✗ | ✗ | ✓ |
| 2. Abdullah et al. 2025 | ✓ | ✓ | ✓ | ✓ | ✗ | ✓ |
| 3. Alsaqqa 2023 | ✗ | ✓ | ✗ | ✗ | ✓ | ✗ |
| 4. Hasan et al. 2024 | ✓ | ✓ | ✗ | ✓ | ✗ | ✓ |
| 5. Mesmeh et al. 2016 | ✗ | ✓ | ✗ | ✓ | ✗ | ✓ |
| 6. Jaradat & Qtait 2025 | ✗ | ✓ | ✗ | ✓ | ✗ | ✓ |
| 7. Bottcher et al. 2019 | ✗ | ✓ | ✓ | ✓ | ✗ | ✓ |
| 8. Albelbeisi et al. 2024 | ✓ | ✓ | ✗ | ✓ | ✗ | ✗ |
| 9. Katchhi & Ming 2025 | ✗ | ✓ | ✓ | ✓ | ✓ | ✓ |

SUMMARY COUNTS:

Theme 1 (Definition and Perception): 3 studies

Theme 2 (Determinants): 9 studies

Theme 3 (Expressions): 4 studies

Theme 4 (Barriers): 6 studies

Theme 5 (Facilitators): 2 studies

Theme 6 (Outcomes): 7 studies

-------------------------------------------------------------------------------

Note: This matrix provides transparency in how individual studies populated the six thematic categories reported in the Results section. Theme definitions are provided in the Synthesis section of the main manuscript.
